# Contemporary data on treatment practices for low-density lipoprotein cholesterol in 6794 patients with stable coronary heart disease across the world

**DOI:** 10.1016/j.dib.2018.04.092

**Published:** 2018-05-01

**Authors:** Anselm K. Gitt, Dominik Lautsch, Jean Ferrières, Gaetano M. De Ferrari, Ami Vyas, Carl A. Baxter, Lori D. Bash, Veronica Ashton, Martin Horack, Wael Almahmeed, Fu-Tien Chiang, Kian Keong Poh, Philippe Brudi, Baishali Ambegaonkar

**Affiliations:** a Herzzentrum Ludwigshafen, Germany; b Institut für Herzinfarktforschung, Ludwigshafen, Germany; c Merck&Co, Inc., Kenilworth, NJ, USA; d Rangueil hospital, Toulouse university school of medicine, Toulouse, France; e Department of Molecular Medicine University of Pavia, and Cardiac Intensive Care Unit and Laboratories for Experimental Cardiology, IRCCS Fondazione Policlinico San Matteo, Pavia, Italy; f Rutgers University, School of Public Health, Piscataway, NJ, USA; g Merck Sharp & Dohme, Corp., Hertford Road, Hoddesdon, UK; h Agile-1 for Merck & Co., Inc., Kenilworth, NJ, USA; i Sheikh Khalifa Medical City, Abu Dhabi, UAE; j Heart and Vascular Institute, Cleveland Clinic, Abu Dhabi, UAE; k National Taiwan University Hospital, Taipei, Taiwan; l Fu-Jen Catholic University Hospital, Taipei, Taiwan; m Yong Loo Lin School of Medicine, National University of Singapore, Singapore; n Department of Cardiology, National University Heart Center Singapore, National University Health System, Singapore

**Keywords:** Low-density lipoprotein cholesterol, Treatment target, Global, Region, Statins

## Abstract

DYSIS II CHD was a longitudinal, observational study in 6794 patients from 18 countries. They were attending an outpatient physician appointment for coronary heart disease (CHD). 6370 patients (93.8%) were on active lipid lowering therapy (LLT). The mean atorvastatin dose equivalent was 25 mg per day and 10.5% received ezetimibe in combination with a statin. The mean low-density lipoprotein cholesterol (LDL-C) level was 88 mg/dL, with 29.4% of patients displaying a level below the 70 mg/dL target for very high-risk subjects.

**Conclusion:**

While more than 90% of patients with CHD were on lipid lowering drugs, only three out of ten patients achieved their LDL-C target value.

**Specifications table**

**Table:** 

Subject area	Biology
More specific subject area	Dyslipidemia and cardiovascular risk
Type of data	Tables and Figures
How data was acquired	Worldwide survey
Data format	Analyzed
Experimental factors	Observational, longitudinal registry
Experimental features	Comparison of lipid lowering therapies administered in patients with coronary heart disease, as well as LDL-C target achievement.
Data source location	Institut für Herzinfarktforschung, Ludwigshafen, Germany
Data accessibility	Data are included in this article

**Value of the data**

• These data have been collected under real life conditions across the world.
• Stratification per country can help to facilitate a scientific dialogue for the benefit of coronary patients in these countries, but also help to compare treatment standards between geographies of the world.
• The data presented can help to guide treatment decisions for novel lipid lowering agents.

## 1 Data

**Fig. 1:**
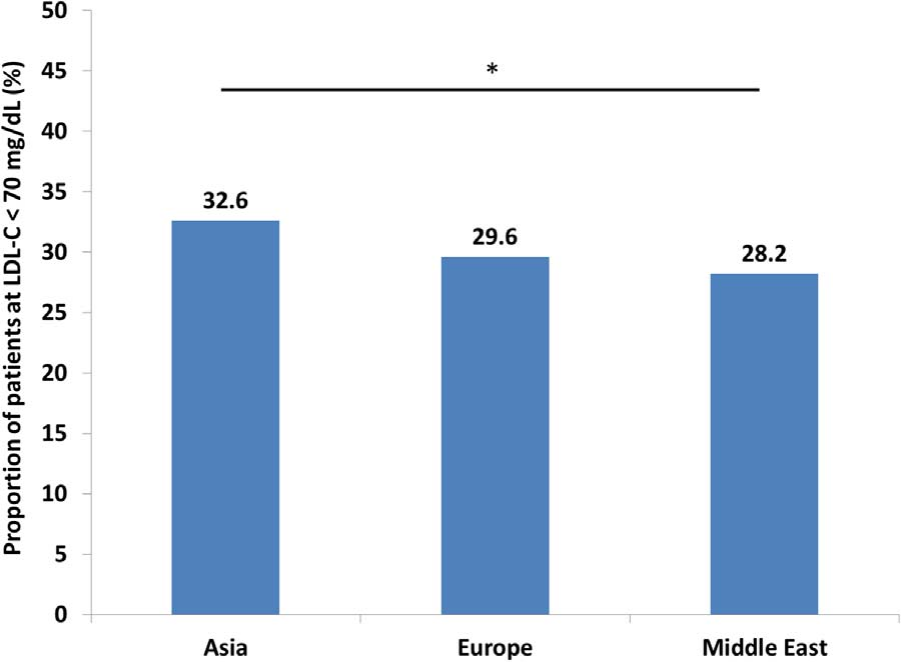
LDL-C target attainment for LLT-treated patients by region Legend: *p<0.05 for overall comparison.

See Fig. 1 and Table 1, Table 2.

**Table 1:** Predictors of LDL-C target value attainment among treated CHD patients.

	Full model			Stepwise model		
	OR	95% CI	*P* value	OR	95% CI	*P* value
Age >70 years	1.09	0.97–1.23	0.166	–	–	–
Female	0.72	0.62–0.83	<0.001	0.72	0.63–0.84	<0.001
BMI >30 kg/m^2^	0.83	0.72–0.95	0.007	0.82	0.71–0.94	0.004
Current smoking	0.82	0.69–0.99	0.035	0.81	0.68–0.97	0.022
Sedentary lifestyle	0.86	0.76–0.97	0.011	0.86	0.76–0.97	0.012
Stable angina	0.88	0.77–0.99	0.041	0.88	0.77–0.99	0.040
Chronic kidney disease	1.20	0.98–1.46	0.076	–	–	–
Type 2 diabetes mellitus	1.70	1.51–1.92	<0.001	1.72	1.53–1.93	<0.001
History of chronic heart failure	0.87	0.73–1.04	0.133	–	–	–
Hypertension	0.81	0.71–0.92	0.001	0.82	0.72–0.93	0.002
Statin dose (>20 mg/day atorvastatin dose equivalent)	1.010	1.007–1.013	<0.001	1.010	1.007–1.013	<0.001

Legend: BMI, body mass index; CI, confidence interval; OR, odds ratio.

**Table 2:** Regional differences in lipid-lowering therapy.

	Asia (*N*=2562)	Europe (*N*=2777)	Middle East (*N*=1031)	*P* value
Statin monotherapy	86.2%	79.8%	79.6%	<0.001
Statin+ezetimibe	7.7%	11.6%	14.6%	<0.001
Statin+other non-statin	5.3%	6.7%	5.0%	0.047
Non-statin monotherapy	0.8%	2.0%	0.7%	<0.001
Atorvastatin dose equivalent (mean±SD mg/day)^a^	20±15	27±20	30±18	<0.0001
Atorvastatin dose equivalent (median [IQR] mg/day)^a^	20 (10, 20)	20 (10, 40)	20 (20, 40)	

SD, standard deviation; IQR, interquartile range.

a In statin treated patients.

## 2 Experimental design, materials and methods

DYSIS II CHD was a multicenter, longitudinal, observational study that included 6794 patients from 18 countries in Europe, the Middle East, South-, Southeast- and East-Asia [1].

The study was approved by the relevant ethics committees and carried out in agreement with local laws.

Inclusion criteria were as follows: 1) provision of written informed consent, 2) aged ≥18, 3) attending an outpatient appointment for stable CHD 2012–2014, 4) availability of a full fasting or non fasting lipid profile from within the previous 12 months, and 5) not participating in a clinical trial.

The ESC/EAS dyslipidemia guidelines (2011) were used as a reference in order to determine target value attainment [2]. Low density lipoprotein (LDL-C) treatment target thus was <70 mg/dl. Use of LLT was documented, including use of statin and combination therapy. We also determined the statin dose administered, calculated as atorvastatin equivalent doses [3].

Data were collected in an electronic case report form and processed in a central web-based database at the Institut für Herzinfarktforschung, Ludwigshafen, Germany. It was used for both collection and storage of the data.

SAS version 9.3 (Cary, NC, USA) was used for performing the calculations. Data are presented as absolute numbers and percentages (*n*/*N*). Between-group differences were evaluated using a chi squared test. Multivariable logistic regression was used to calculate odds ratios for factors predictive of LDL-C target attainment. Both the results of the full model and a stepwise forward selection model are given.
